# Optimising the diagnostic accuracy of First post-contrAst SubtracTed breast MRI (FAST MRI) through interpretation-training: a multicentre e-learning study, mapping the learning curve of NHS Breast Screening Programme (NHSBSP) mammogram readers using an enriched dataset

**DOI:** 10.1186/s13058-024-01846-1

**Published:** 2024-05-28

**Authors:** Lyn I. Jones, Andrea Marshall, Rebecca Geach, Premkumar Elangovan, Elizabeth O’Flynn, Tony Timlin, Sadie McKeown-Keegan, Janice Rose, Sarah Vinnicombe, Sian Taylor-Phillips, Mark Halling-Brown, Janet A. Dunn, the FAST MRI Study Group, Clare Alison, Karen Atkinson, Miklos Barta, Gemini Beckett, Claudia Betancourt, Julie Bramwell, Holly Brown, Helen Burt, Louise Cann, Nick Carter, Claire Cartledge, Jane Ceney, Gillian Clark, Eleanor Cornford, Elizabeth Cullimore, Siân Curtis, Diana Dalgliesh, Jonathon Delve, Sarah Doyle, Alison Duncan, Holly Elbert, Sarah Fearn, Christopher Foy, Zsolt Friedrich, Hesam Ghiasvand, John Gifford, Dagmar Godden, Zoe Goldthorpe, Sandra Gomes, Narayan Aradhana Goud, Rosie Gray, Sam A. Harding, Kristin Henning, Lucinda Hobson, Claire Hulme, Paula Hynam, El Sanharawi Imane, Emma Jackson, Asif Jaffa, Ragini Jhalla, Margaret Jenkin, Thomas William Jones, Nahid Kamangari, Vandana Kaur, Beckie Kingsnorth, Katherine Klimczak, Elisabeth Kutt, Karen Litton, Simon Lloyd, Iain Lyburn, Anjum Mahatma, Anna Mankelow, Helen Massey, Helen Matthews, Karis McFeely, Clare McLachlan, Sarah McWilliams, Shahrooz Mohammadi, Alice Moody, Elizabeth Muscat, Sreenivas Muthyala, Sarah Perrin, Alison Peters, Alice Pocklington, Elizabeth Preston, Jasvinder Rai, Jo Robson, Corri Salter, Toni Scanlon, Anuma Shrestha, Richard Sidebottom, Mary Sinclair, Sravya Singamaneni, Jim Steel, Lesley Stephenson, Sam Stewart-Maggs, Cheryl Stubbs, Michelle Taylor, Victoria Taylor, Olivia Taylor-Fry, Erika Toth, Matthew Trumble, Alexandra Valencia, Frances Vincent, Anna Wang, Lucy Warren, Sharon Watkin, Sue Widdison, Jennifer Williams, Jennifer Wookey

**Affiliations:** 1 North Bristol NHS Trust, Southmead Hospital, Southmead Road, Westbury on Trym, Bristol, BS10 5NB UK; 2 Warwick Clinical Trials Unit, University of Warwick, Coventry, CV4 7AL UK; 3 Scientific Computing Department, Royal Surrey NHS Foundation Trust, Guildford, Surrey GU2 7XX UK; 4 St George’s University Hospitals Foundation Trust, London, SW17 0QT UK; 5 Independent Cancer Patients’ Voice, London, EC1R 0LL UK; 6 Gloucestershire Hospitals NHS Foundation Trust, Cheltenham, GL53 7AS UK

**Keywords:** FAST MRI, Abbreviated breast MRI, Breast cancer, Screening, Formative assessment, Medical education, Diagnostic accuracy, e-learning

## Abstract

**Background:**

Abbreviated breast MRI (FAST MRI) is being introduced into clinical practice to screen women with mammographically dense breasts or with a personal history of breast cancer. This study aimed to optimise diagnostic accuracy through the adaptation of interpretation-training.

**Methods:**

A FAST MRI interpretation-training programme (short presentations and guided hands-on workstation teaching) was adapted to provide additional training during the assessment task (interpretation of an enriched dataset of 125 FAST MRI scans) by giving readers feedback about the true outcome of each scan immediately after each scan was interpreted (formative assessment). Reader interaction with the FAST MRI scans used developed software (RiViewer) that recorded reader opinions and reading times for each scan. The training programme was additionally adapted for remote e-learning delivery.

**Study design:**

Prospective, blinded interpretation of an enriched dataset by multiple readers.

**Results:**

43 mammogram readers completed the training, 22 who interpreted breast MRI in their clinical role (Group 1) and 21 who did not (Group 2).

Overall sensitivity was 83% (95%CI 81–84%; 1994/2408), specificity 94% (95%CI 93–94%; 7806/8338), readers’ agreement with the true outcome kappa = 0.75 (95%CI 0.74–0.77) and diagnostic odds ratio = 70.67 (95%CI 61.59–81.09). Group 1 readers showed similar sensitivity (84%) to Group 2 (82% *p* = 0.14), but slightly higher specificity (94% v. 93%, *p* = 0.001). Concordance with the ground truth increased significantly with the number of FAST MRI scans read through the formative assessment task (*p* = 0.002) but by differing amounts depending on whether or not a reader had previously attended FAST MRI training (interaction *p* = 0.02). Concordance with the ground truth was significantly associated with reading batch size (*p* = 0.02), tending to worsen when more than 50 scans were read per batch. Group 1 took a median of 56 seconds (range 8–47,466) to interpret each FAST MRI scan compared with 78 (14–22,830, p < 0.0001) for Group 2.

**Conclusions:**

Provision of immediate feedback to mammogram readers during the assessment test set reading task increased specificity for FAST MRI interpretation and achieved high diagnostic accuracy. Optimal reading-batch size for FAST MRI was 50 reads per batch.

*Trial registration* (25/09/2019)**:** ISRCTN16624917.

**Supplementary Information:**

The online version contains supplementary material available at 10.1186/s13058-024-01846-1.

## Background

Screening with breast MRI can reduce interval cancers for women with very dense breasts but otherwise at population-risk of breast cancer [1, 2]. First post-contrAst SubtracTed MRI (FAST MRI), a shortened form of breast MRI, has been reported to retain the sensitivity for aggressive breast cancer of full protocol breast MRI (fpMRI) [3, 4], and its shorter acquisition and interpretation times make it more likely to be cost-effective [5]. The diagnostic accuracy of FAST MRI has been shown to be similar to that of fpMRI when reported by experts in fpMRI interpretation [6, 7]. FAST MRI is being introduced into clinical practice to screen a wider group of women than those currently screened with fpMRI [8, 9].

Internationally, many fewer radiologists interpret fpMRI than interpret screening mammograms [10]. Published expert opinion on the implementation of FAST MRI into screening practice has emphasized the importance of performance audit for readers whilst suggesting that benchmarks for interpretation can be developed following roll out without specific interpretation-training for existing fpMRI readers [8]. However, by excluding mammogram readers who do not currently interpret fpMRI from FAST MRI interpretation, this approach could limit the potential expansion of the role of FAST MRI by limiting the numbers of radiologists who may interpret it. For FAST MRI to be scaled up within screening programmes worldwide, effective FAST MRI interpretation-training for mammogram readers is needed.

The Society of Breast MRI provided interpretation-training to experienced fpMRI readers prior to their interpretation of FAST MRI within the EA1141 breast screening trial [3]. No formal evaluation of this training was published but diagnostic accuracy achieved at single read within this trial for FAST MRI was 96% sensitivity and 87% specificity [3].

The authors’ own FAST MRI Study Group previously published a multi-centre study evaluating the effectiveness of FAST MRI interpretation-training [11]. This study built on earlier work to develop a standardised training programme for NHS Breast Screening Programme (NHSBSP) mammogram readers [12, 13]. Following a single day’s training, mammogram readers achieved an overall sensitivity of 86% and specificity of 86%. However, the diagnostic accuracy achieved in the study by those with previous experience of reporting fpMRI (sensitivity 89%, specificity 90%) remained higher than for those with no such previous experience (sensitivity 83% *p* < 0.001, specificity 82% *p* < 0.001) [11]. We could find no other publications that evaluated the effectiveness of FAST MRI interpretation-training.

Formative assessment is an educational technique where the assessment process includes feedback to the learner so that in addition to measuring the learner’s achievement, it enhances learning [14]. We hypothesised that readers’ diagnostic accuracy could be optimised by converting summative assessment (without any feedback to the readers), as used in previous FAST MRI reader training studies [11, 13] into formative assessment (by giving readers immediate feedback for each FAST MRI scan read during the assessment task).

The aims of the study were:

- To determine whether mammogram readers’ diagnostic accuracy can be improved through the addition of formative assessment [14] to standardised FAST MRI interpretation-training
- To map the learning curve for NHSBSP mammogram readers during FAST MRI interpretation-training (by evaluating the incremental diagnostic accuracy of both experienced and novice MRI readers during the formative assessment process).

## Methods

This study was reviewed and approved by the Health and Care Research Wales Ethics Committee and by the Health Research Authority (REC:21/HRA/4543 IRAS:301,714) and prospectively registered (ISRCTN:16,624,917) and all participants gave written informed consent.

### Study design

Prospective, blinded interpretation of an enriched dataset by multiple readers.

### Participants and setting

All NHSBSP mammogram readers who were fully qualified to interpret mammograms, at 7 sites in England were invited to take part (December 2021—February 2022) and were classified as Group 1 if they also interpreted fpMRI in their normal clinical practice, and Group 2 if not. Both Groups were sub-classified into those who had previously undertaken in-person FAST MRI training as part of a research study (“Attended”) and those that had not (“Not attended”). Participants then independently completed two days of standardised FAST MRI interpretation-training, of which the second day comprised reading a test set of FAST MRI scans with feedback on the true outcome for each scan being given immediately after their opinion was recorded (formative assessment [14]). The training was undertaken remotely at times chosen by the readers (January—June 2022).

### Standardised training

A previously developed standardised training programme [11–13] (details reproduced in Additional file 1), was adapted to enable remote self-directed independent e-learning by participants. Previously developed small-group presentations and hands-on workstation sessions were recorded and made available to participants online, as videos. Additionally, software was provided to the NHS sites that enabled learners to simultaneously login to a software platform (RiViewer) on NHS workstations to practice image manipulation of 29 FAST MRI scans (the training set), guided by the recorded sessions.

Readers were taught how to classify FAST MRI scans according to the UK 5-point breast imaging classification specified for screening fpMRI in women at higher risk of breast cancer within NHSBSP [15]. When adding a point region of interest (ROI) to an image they were prompted to label the ROI with an MRI classification from the UK 5-point scale. Quantification of the UK 5-point scale, defining how it maps to the BI-RADS classification system was described by Taylor et al. [16].

Following the standardised training, readers were instructed to read the test set of 125 FAST MRI scans (see below). In the current study, the new provision of immediate feedback (on the true outcome of each scan) during the test set reading assessment task (termed formative assessment [14]) formed a new and additional part of the reader training.

### Test set

The test set comprised 125 FAST MRI scans with known outcome, acquired as fpMRI during 2015, including a consecutive high-risk screening series (72 scans) enriched with additional cancer cases from the same year (53 scans). All cancer cases had histological confirmation and non-cancer scans were confirmed with two-year follow-up. Details of this test set have been described previously [11, 13] (FAST MRI specification and test set composition have been reproduced in Additional file 2). Of 125 FAST MRIs in the test set, 54 had biopsy-confirmed unilateral cancer and one bilateral (56 breasts with cancer) and 2 women had two separate tumours identified in the same breast, giving a total of 58 cancers reported in the ground truth, 56 invasive and 2 ductal carcinoma-in-situ (DCIS).

The current study used the same test set as the previous interpretation-training studies [11, 13]. The training in the current study differed from that delivered previously in being delivered as remote e-learning and in the method of delivery of the assessment being as formative assessment. Participants who had taken part in a previous interpretation-training study (11 in Group 1 and 7 in Group 2) were viewing the FAST MRI scans of the test set for the second time. However, prior to the start of the current study, the ground truth (true outcome) of the test set had at no time been revealed to them and the average time interval between reading the test set in one of the two previous studies and in the current study was 24 months (range 17 – 30 months).

### Electronic format

Previously developed software (RiViewer), that displays FAST MRI as maximum intensity projection (MIP) and stacked, subtracted slices, was used in which biopsy-proven cancers had been drawn onto images electronically as regions of interest (ROI volumes) to provide ground truth. As in a previous study [11], during guided hands-on workstation training, learners reviewed the training set of 29 FAST MRI scans and could discover the ground truth at the touch of a button.

**Fig. 1:**
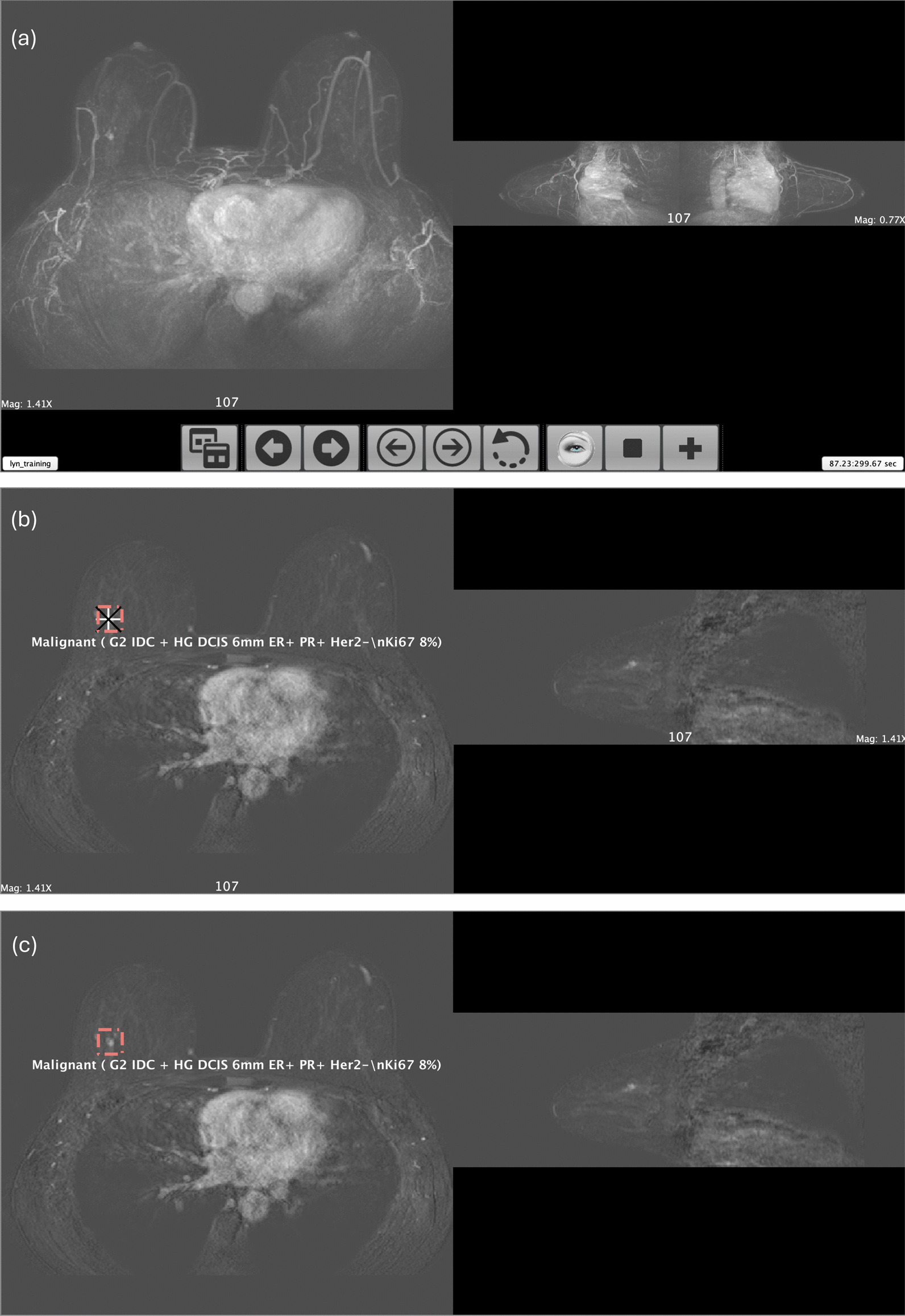
RiViewer software display of an example cancer case from the test set **a** Maximum intensity projections (MIP). The whole RiViewer screen is shown, including the control panel and timer. **b** Axial and sagittal slices as they would appear to a reader who has correctly identified a cancer and committed to their opinion for the case. The ground truth is displayed as a red, volume region of interest (ROI) and the reader’s point ROI is displayed as white and black superimposed crosses. Pop-up white text describes the ground truth/cancer histology. **c** Axial and sagittal slices with the reader’s point ROI hidden to enable the reader to review the cancer appearance prior to moving on to view the next scan. This figure demonstrates how the software enabled trainee readers to compare their own opinion with the ground truth of each scan during formative assessment in the current study

In the current study the software was adapted so that the test set of 125 FAST MRI scans was presented as formative assessment (providing a second, additional day of training). During test set interpretation in the current study, once participants had completed their interpretation of each scan and committed their assessment electronically, it was automatically locked in and they were immediately able to view the ground truth of the scan, superimposed on their own opinion. This gave them instant feedback prior to their viewing of the next scan in the test set. Figure 1 presents an example cancer case from the test set and demonstrates how, during the current study’s formative assessment task, the RiViewer software enabled trainee readers to compare their own opinion with the ground truth of each scan.

Training and test set MRIs are mutually exclusive and were from a single centre but acquired during different years, from different women.

The readers’ point ROI needed to be classified as MRI 4 or 5 by the reader and to be sited within the ground truth ROI volume of a histologically proven cancer for their opinion to be registered as having correctly detected a cancer.

The RiViewer software includes an automatic timer which recorded the time taken by each reader to interact with each scan.

### Test set interpretation

Having completed the training set, participants interpreted the test set of 125 FAST MRIs, blinded to all other information (clinical history, previous imaging, histology, and other readers’ interpretations). Readers were told to expect more cancers than in usual screening practice but no other indication of the number of cancers was given. The test set was presented to each reader in a different random order.

For the current study, readers were encouraged to complete their reading of the assessment test set (formative assessment task) within as short a time as was reasonably possible, following completion of the other training material. No recommendations were made regarding the number of scans to be read at a time (batch size).

### Sample size calculation

Using the results of a previous interpretation-training study [13], a sample of 250 breasts from 125 women would allow the lower 95% confidence limit of the inter-rater reliability (Kappa statistic) to be estimated to within 0.07 with a minimum of 6 readers in each group and a proportion of cancers of 0.22 [17]. Thus, to assess inter-rater reliability, we required a minimum total of 12 readers: 6 in each group.

### Statistical analysis

Per-breast analysis of the frequency of results against true outcome was obtained overall and for each reader. Sensitivity, specificity, and concordance of readers’ FAST MRI classification with the true outcome were determined and differences across reader groups and previous attendance on a FAST MRI training session assessed using a multi-level-generalised-mixed model to account for multiple readers per scan and the dependence between breasts. Restricted cubic splines with 4 knots to the number of FAST MRI scans read overall and per reading session (batch size) were also included in the models to assess whether the readers performance improved during the assessment task.

The agreement between readers and the true outcome was assessed using Cohen’s κ coefficient, to account for the probability of agreement occurring by chance. The diagnostic odds ratio was determined as a measure of overall diagnostic accuracy independent of prevalence [18]. Classifications 4 and 5 were considered indicative of cancer, and classifications 1–3 considered a normal result.

Interpretation times were compared across reader groups (Wilcoxon rank-sum).

Sensitivity and specificity from the current study were compared with previous FAST MRI training results from the two studies that used in-person versions of the same standardised training programme and assessment dataset (delivered as one-to-one [13] or small group [11] training) using a bivariate random effect model to account for the dependency between sensitivity and specificity.

## Results

There were 43 participants from 7 sites, 22 with previous experience of reading fpMRI (Group 1) and 21 new to reading MRI (Group 2). Eighteen participants (11 from Group 1 and 7 from Group 2) had previously undertaken in-person FAST MRI training as part of a research study (“Attended”) and the remaining participants in each group had not, (“Not attended”) [11, 13].

All participants completed the current training, including reading the formative assessment task (test set) of 125 FAST MRI scans (250 breasts). Individual readers’ opinions for 4 scans failed to register due to a technical error, giving a total of 10,746 reads.

**Fig. 2:**
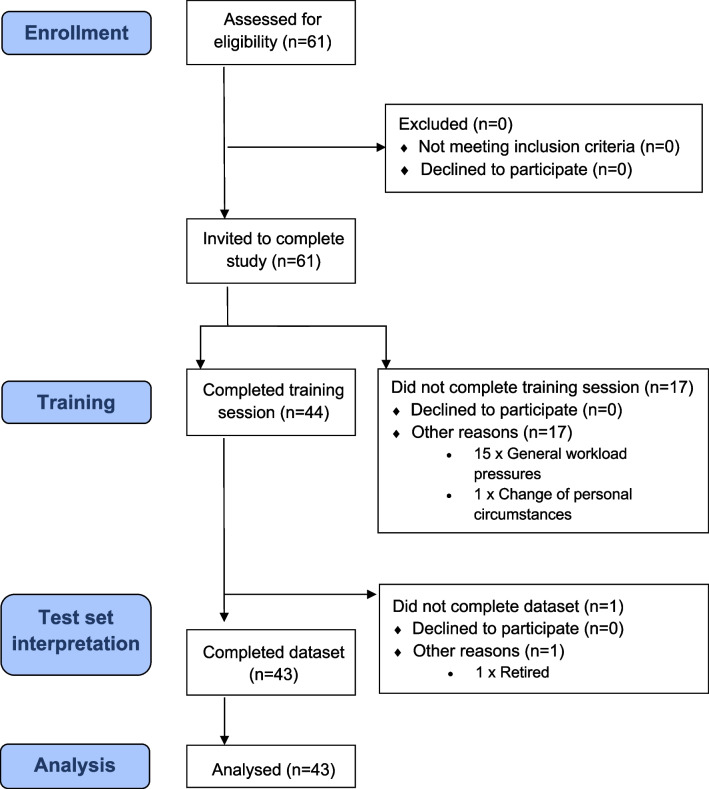
Flow diagram detailing participation in FAST MRI interpretation e-learning study

Figure 2 shows the flow chart of reader recruitment (Fig. 2) and Table 1 details participants’ professional experience (Table 1).

**Table 1:** Demographics of participant mammogram readers

	Group 1*	Group 2*
Number of participants	22	21
Professional Title**		
Advanced Practitioner	0	7
Consultant Radiographer	0	7
Breast Clinician	0	4
Radiologist Associate Specialist	0	1
Consultant Radiologist	22	2
**Professional Experience**		
Number of years interpreting mammograms: median (range)	9 (1–22)	7 (1–28)
Number of mammograms interpreted each year: median (range)	6000 (3000–12000)	7000 (4000–10000)
Participant readers who interpret digital breast tomosynthesis (DBT) in normal clinical practice	22	15
Number of years interpreting breast MRI: median (range)	8 (1–25)	N/A
Number of full protocol breast MRI scans interpreted each year: median (range)	135 (30–450)	N/A
Total numbers of participant readers who previously attended in person FAST MRI training	11	7

^*^Reader group: group 1 = mammogram readers with experience of fpMRI interpretation in their usual clinical practice, group 2 = mammogram readers with no previous experience of breast MRI interpretation in their clinical practice

^**^Professional titles in UK: Screening mammograms within the NHS Breast Screening Programme are interpreted by multidisciplinary healthcare professionals trained in mammogram interpretation. Their performance is subject to continuous audit through the UK Breast Screening Information System that produces individual real-life performance data over rolling 3-year periods [38]

“Consultant Radiologist” and “Breast Clinician” are titles held by medical doctors. Consultant Radiologists are registered on the General Medical Council’s Specialist Register following Completion of Specialist Training (5 years) with standards and curriculum set by the Royal College of Radiologists (RCR). The Association of Breast Clinicians launched the Credential in Breast Disease Management for Breast Clinicians, jointly with the RCR, in 2019, to standardise and formalise training for Breast Clinicians across the UK (3-year training programme) [39]

“Advanced Practitioners” and “Consultant Radiographers” are experienced, registered healthcare practitioners, typically mammographers, who have additionally completed specialist training, underpinned by a master’s level award or equivalent to support their professional practice within the NHS [40]

### Per-breast analysis

The per-breast analysis comparing readers’ MRI classification with the true outcome (cancer or normal) showed an overall sensitivity of 83% (95%CI 81–84%; 1994/2408) and specificity of 94% (95%CI 93–94%; 7806/8338).

Readers with experience of fpMRI interpretation (Group 1) showed similar sensitivity (1034/1232; 84%; 95%CI 82–86%) but slightly higher specificity (4031/4266; 94%; 95%CI 94–95%) than readers without fpMRI experience (Group 2) (sensitivity = 82%; 95%CI 79–84% (960/1176) *p* = 0.14; specificity = 93%; 95%CI 92–93% (3775/4072) *p* = 0.001) (Table 2).

**Table 2:** Diagnostic accuracy of readers by group* and by attendance or non-attendance at previous in person FAST MRI interpretation-training

Category	Measure				
	Concordance (Accuracy)	True positive rate (Sensitivity)	True negative rate (Specificity)	Kappa (95% CI)	Diagnostic odds ratio (95% CI)
All readers	9800/10746 (91%)	1994/2408 (83%)	7806/8338 (94%)	0.75 (0.74–0.77)	70.67 (61.59–81.09)
Reader group*					
Group 1*	5065/5498 (92%)	1034/1232 (84%)	4031/4266 (94%)	0.77 (0.76–0.80)	89.58 (73.26–109.52)
Group 2*	4735/5248 (90%)	960/1176 (82%)	3775/4072 (93%)	0.73 (0.70–0.75)	56.49 (46.76–68.25)
*Attendance at previous in person FAST MRI interpretation training*					
Attended	4123/4500 (92%)	876/1008 (87%)	3247/3492 (93%)	0.77 (0.75–0.79)	87.95 (70.27–110.08)
Not attended	5677/6246 (91%)	1118/1400 (80%)	4559/4846 (94%)	0.74 (0.72–0.76)	62.98 (52.77–75.16)
Reader group* and attendance at previous in person FAST MRI interpretation training					
Group 1* Attended	1613/1750 (92%)	533/616 (87%)	2002/2134 (94%)	0.78 (0.75–0.81)	97.40 (72.83–130.25)
Group 1* Not attended	2530/2748 (92%)	501/616 (81%)	2029/2132 (95%)	0.77 (0.74–0.80)	85.82 (64.65–113.93)
Group 2* Attended	1588/1750 (91%)	343/392 (88%)	1245/1358 (92%)	0.75 (0.71–0.78)	77.12 (54.03–110.09)
Group 2* Not attended	3147/3498 (90%)	617/784 (79%)	2530/2714 (93%)	0.71 (0.69–0.74)	50.80 (40.48–63.76)

^*^ Reader group: group 1 = experience of fpMRI interpretation in their usual clinical practice, group 2 = no previous experience of breast MRI interpretation in their clinical practice

**Fig. 3:**
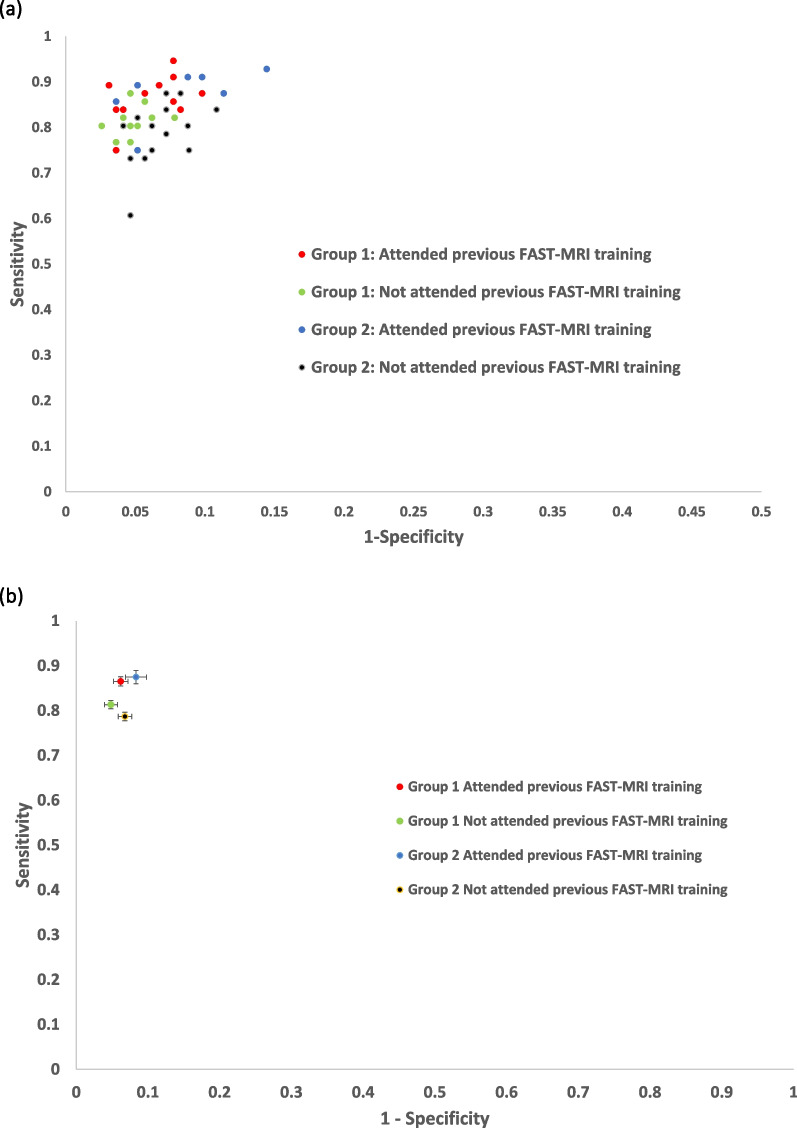
Diagnostic accuracy in the receiver operating characteristic (ROC) space **a** Point estimates of accuracy for individual readers in ROC space **b** Plot of accuracy in ROC space for each group and attendance or non-attendance with error bars for 95% Confidence Intervals (95%CIs)

Those readers that had previously completed in-person FAST MRI interpretation training (“Attended”) had a significantly higher overall sensitivity (88%; 95% CI 85–91%) than those that had not attended (80%; 95% CI 78–82%, *p* < 0.0001), but significantly lower specificity (92%; 95% CI 92–93% compared to 94%; 95% CI 94–95%, *p* = 0.003), irrespective of group (Table 2). The diagnostic accuracy results are summarised in Fig. 2, which plots readers’ accuracy in the receiver operating characteristic space by group and by whether previously attended in person FAST MRI training (Fig. 3).

Both the inter-reader agreement (kappa) of readers with the true outcome and the diagnostic odds ratio (DOR) were higher for Group 1 (kappa 0.77 (95%CI: 0.76–0.80), DOR 89.58 (95% CI 73.26–109.52) than Group 2 (0.73 (0.70–0.75) and 56.49 (46.76–68.25)) and tended to be higher for those participants that had attended previous FAST MRI training (kappa 0.77 (95%CI: 0.75–0.79), DOR 87.95 (95% CI 70.27–110.08) compared to those participants that had not previously completed FAST MRI training (kappa 0.74 (95%CI: 0.72–0.76), DOR 62.98 (95% CI 52.77–75.16) (Table 2).

### Plotting the learning curve

Readers’ sensitivity remained fairly stable during the test set reading process (formative assessment task) (p = 0.24) and this effect was similar for both groups (interaction *p* = 0.30) and whether or not they had previously completed FAST MRI training (interaction *p* = 0.97).

**Fig. 4:**
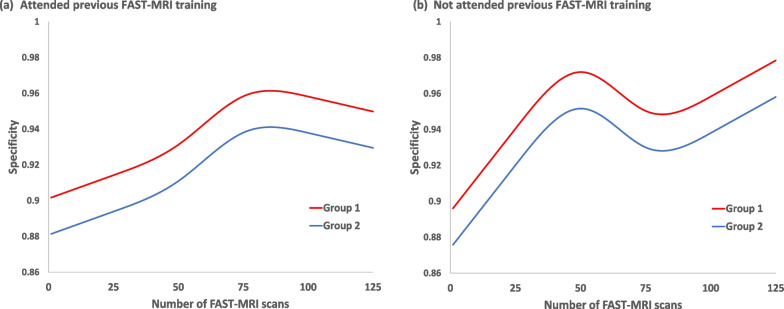
Changes in reader specificity with number of test-set FAST MRI scans read over time Multi-level generalised mixed model using restricted cubic splines with 4 knots to the number of scans read over time by attendance or non-attendance at previous FAST-MRI training and by group

However, specificity was significantly affected by the number of scans read in the formative assessment task (*p* < 0.001) and this effect differed, depending on whether readers had attended previous FAST MRI training or not (interaction *p* = 0.01) but not between groups (interaction *p* = 0.08). The predicted specificity curves for readers that had attended previous FAST MRI training reached a peak after 75 reads but continued to increase for those that had not attended previous FAST MRI training, with group 1 readers having significantly higher specificity than group 2 (*p* = 0.003) (Fig. 4).

**Fig. 5:**
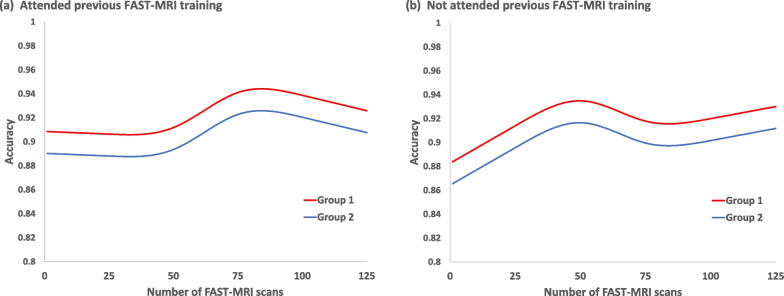
Changes in concordance with the true outcome by number of test-set FAST-MRIs read over time Multi-level generalised mixed model using restricted cubic splines with 4 knots to the number of scans read over time by attendance or non-attendance at previous FAST-MRI training and by group

Accuracy (concordance with the true outcome) changed significantly by the number of FAST MRI scans read in the test set reading process (formative assessment task) (*p* = 0.002) and the change differed depending on whether or not a reader had previously attended FAST MRI training (interaction *p* = 0.02) but was similar for both groups (interaction *p* = 0.36). Accuracy was significantly higher for Group 1 than Group 2 overall (*p* = 0.001) and reached a peak after 75 reads for those readers that had previously attended FAST MRI training, as seen with the results for specificity (Fig. 5).

### Reading pattern: batch size

Readers read the formative assessment task of 125 FAST MRI scans in a median of 2 batches (range 1- 8 batches) with a median of 32 scans read per batch (range 1–125 scans/batch). The readers that did not attend the previous FAST MRI in person training tended to complete this current training in fewer batches and hence had larger batch sizes than those that had attended previous training (Table 3).

**Table 3:** Batch reading pattern by group* and by attendance or non-attendance at previous in person FAST MRI interpretation-training

	Group 1*	Group 1*	Group 2*	Group 2*
	Attended	Not attended	Attended	Not attended
*Number of batches*				
Median (IQR)	2 (2–4)	2 (1–4)	3 (3–5)	1 (1–3)
Range	1–8	1–5	2–8	1–7
*Number of FAST MRI scans read in a batch*				
Median (IQR)	31 (15–58)	33 (23–63)	30 (14–41)	50 (19–125)
Range	9–125	7–125	1–84	1–125

^*^ Reader group: group 1 = experience of fpMRI interpretation in their usual clinical practice, group 2 = no previous experience of breast MRI interpretation in their clinical practice

**Fig. 6:**
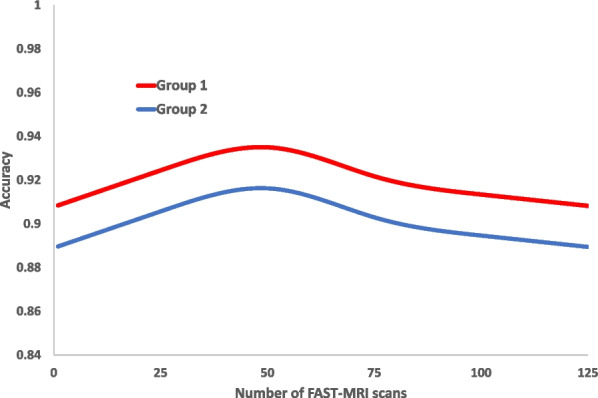
Changes in concordance with the true outcome by number of test-set FAST-MRIs read per batch Multi-level generalised mixed model using restricted cubic splines with 4 knots fitted to the rank order of FAST-MRI scans read per batch by reader group

Accuracy (concordance with the true outcome) significantly changed depending on the number of reads within a batch (*p* = 0.02) but in a similar manner for both groups (interaction *p* = 0.53) and for whether or not a reader had previously attended FAST MRI training (interaction *p* = 0.78). Accuracy tended to worsen after 50 FAST MRI scans were read within a batch for both groups (Fig. 6).

Similarly, specificity significantly changed with the number of reads within a batch (*p* = 0.0001) for both groups (interaction *p* = 0.18). Sensitivity, although not significantly dependent, tended to worsen with increasing number of MRI scans read within a batch (*p* = 0.08) and this was similar for both groups (interaction *p* = 0.91).

### Time taken to interpret

The median time taken for the individual readers to interpret each FAST MRI scan was 22 s less for Group 1 (median 56 s, range 8–47,466 s) than for Group 2 (median 78 s, 14–22,830 s, p < 0.0001). Seven records had a total time of more than 1000 s.

### Time taken to train

The time taken by each reader to complete the training (standardised e-learning programme) comprised time spent watching recorded presentations (180 min), interacting with training scans guided by recorded hands-on workstation training videos (median 56 min, range 5–195) and interacting with the assessment test set as formative assessment (173 min, range 58–977). The median total time that a reader took to train was 411 min (range 257–1209) = 7 h (4–20). Additional, unquantifiable time would have been needed to log in and out of the web portals and to take breaks as required during training.

### Comparison with previous FAST MRI training results

FAST MRI readers in the current study (trained with remote e-learning and automated formative assessment) achieved significantly higher specificity (94%; 95%CI 93–94) than in the one-to-one training [13] (87%; 95% CI 85–89, *p* < 0.001) and in the small group training [11] (86%; 95% CI 85–86, *P* < 0.001) (Table 4). There was, however, lower sensitivity (83%; 95% CI 81–84) compared to the one-to-one training [13] (88%; 95% CI 84–91, p = 0.01) and the small group training [11] (86%; 95% CI 84–87, *p* = 0.008). Overall, the concordance with the true outcome, reader agreement with the true outcome (kappa) and the DOR achieved by readers were all higher in the current study than in the previous two studies that used the in-person versions of the same training programme and assessment dataset (Table 4).

**Table 4:** Diagnostic accuracy within the current study in comparison to previous training results from the two studies that used in-person versions of the same standardised training programme and assessment dataset (delivered as one-to-one(13) or small group(11) training)

Category	Measure				
	True positive rate (Sensitivity) (95%CI)	True negative rate (Specificity) (95%CI)	Concordance with the true outcome (Accuracy) (95% CI)	Kappa (95% CI)	Diagnostic odds ratio (95% CI)
Current Study: All readers	83% (81–84)	94% (93–94)	91% (91–92)	0.75 (0.74–0.77)	70.67 (61.59–81.09)
One to one training (13)	88% (84–91)	87% (85–89)	87% (86–89)	0.69 (0.65–0.72)	48.30 (35.12–66.44)
Small group training (11)	86% (84–87)	86% (85–86)	86% (85–87)	0.63 (0.61–0.65)	35.49 (30.87–40.81)

## Discussion

Training within the current study, using remote e-learning and automated formative assessment, improved overall diagnostic accuracy (concordance with the true outcome, reader agreement with the true outcome (kappa), and DOR) and specificity compared to previous FAST MRI training using in-person versions of the same standardised training programme and assessment dataset delivered as one-to-one [13] or small group [11] training. There was, however, lower sensitivity at cancer detection.

Learning curves of increasing overall accuracy (concordance with the true outcome) and of increasing specificity were observed during the formative assessment task but reader sensitivity did not significantly change, and this was observed for all categories of reader. Those who had attended previous FAST MRI interpretation-training reached peak overall accuracy and specificity at 75 scans read but for those new to FAST MRI interpretation, specificity continued to increase.

Maximising the overall diagnostic accuracy of a test is desirable but, for a given overall diagnostic accuracy, there is a trade-off between the test’s sensitivity and specificity. For a diagnostic imaging test, interpretation-training provides an opportunity to improve overall diagnostic accuracy and can also be used to influence the balance between reader sensitivity and specificity. The choice of which metric (sensitivity or specificity) is more important greatly depends on the pre-test probability of the population to be screened. For example, the survival benefit achieved, through screening women with BRCA mutations (high pre-test probability), using fpMRI, is dependent on fpMRI’s high sensitivity for aggressive breast cancers and necessitates the prioritisation of sensitivity over specificity for this relatively small population of women [19–21]. In contrast, the specificity for mammographic mass screening that is achieved through double reading in the NHS Breast Screening Programme (NHSBSP), is 96% [22] while reported reader sensitivity is much lower (67–78%) [23]. For population-risk women, who have a low pre-test probability, specificity is arguably the most important diagnostic accuracy parameter to optimise because small changes in specificity can have a large effect on the number of false positive recalls in a population screening programme, with each recall causing harm to the woman screened and also incurring a financial and workforce cost [24–26].

FAST MRI was designed as a screening test that would provide a higher sensitivity for aggressive breast cancers than mammography at a fraction of the cost of fpMRI, through shorter acquisition and reading times [4], with the intention that it could be used to screen a wider population than currently benefit from screening with fpMRI [19, 20]. Trials of breast MRI (scans single read by expert fpMRI readers) for women with dense breasts, but otherwise at population risk of breast cancer, have reported results with high sensitivity (95.7% [3] and 95.2% [1]) but lower specificity (86.7% [3] and 92.6% [1]). If FAST MRI is to be provided at scale to a large population of women with low pre-test probability, then both specificity-optimisation and expansion of the workforce of MRI readers will be required. The specificity achieved for FAST MRI by mammogram readers in the current study following 2 days of standardised training (94%) compares well with the results from both these reported MRI screening trials and approaches the specificity of mammography achieved with double reading within the NHSBSP for population screening (96%) [22].

In the current study, readers achieved, at single read, a sensitivity of 83% in a challenging dataset that included a high proportion of lobular carcinomas and of mammographically occult cancers and an invasive cancer size ≤ 25 mm (Additional file 2) [13]. Whilst this level of sensitivity could be considered insufficient to screen a population at very high risk of breast cancer, it could potentially be increased through double reading [27], and could be adequate to screen a larger population with lower pre-test probability, given the significant gains achieved in specificity and overall diagnostic accuracy.

### Achievement of reporting benchmarks for fpMRI and literature comparison of diagnostic accuracy

Two days of standardised FAST MRI interpretation-training, undertaken as remote e-learning, enabled NHSBSP mammogram readers, both those experienced in fpMRI interpretation (Group 1) and novice MRI readers (Group 2), to achieve, at single read of an enriched dataset, benchmarks set for fpMRI interpretation in practice by the American College of Radiology’s Breast Imaging Reporting and Data System (BI-RADS) for both sensitivity (Groups 1 (84%) and 2 (82%) vs. > 80% BI-RADS benchmark [28]) and specificity (Groups 1 (94%) and 2 (93%) vs. > 85% BI-RADS benchmark [28]). Of 43 participants, the two-day remote e-learning programme was sufficient for 43/43 (100%) to achieve specificity above the 85% BI-RADS benchmark and for 33/43 (77%) to achieve sensitivity above the 80% BI-RADS benchmark.

Novice MRI readers (Group 2) achieved similar sensitivity to experienced fpMRI readers (Group 1) (*p* = 0.14) but lower specificity (*p* = 0.001) although specificity differed between groups by only one percentage point (Group 1: 94% and Group 2: 93%).

The single reading performance at FAST MRI achieved by experienced (Group 1) and novice (Group 2) readers in the current study, reading an enriched dataset, compares well with published figures for diagnostic performance at fpMRI for radiologists experienced in breast MRI interpretation in community screening practice in the USA (Breast Cancer Surveillance Consortium (BCSC) [29]: sensitivity: 84% (Group 1) and 82% (Group 2) vs. 81% (BCSC), and specificity: 94% (Group 1) and 93% (Group 2) vs. 83% (BCSC).

### Comparison between the performance of those who had previously attended in-person FAST MRI interpretation training and those who had not

Whilst the reader agreement with the true outcome (kappa) and the DOR did not differ significantly between the readers who had previously attended in-person FAST MRI interpretation-training (11/22 in Group 1 and 7/21 in Group 2) and those who had not, the sensitivity for cancer detection was higher and the specificity lower for the “attended” cohort than for the “not attended” cohort. Looking at the individual performance, during a previous study [11], of the 14 participants of the current study who had attended previous small group training, 8 of these participants had a sensitivity in the top 9 sensitivities of participants in the previous study and none were in the bottom 7 sensitivities [11]. Additionally, 8 of these participants had specificity in the bottom 12 for specificity in the previous study and 3 were in the top 11 specificities [11]. Therefore, self-selection bias could have contributed to the within group significant differences of sensitivity and specificity found for attendance vs. non-attendance at previous in person training.

### Literature comparison – the effect of batch size on diagnostic performance

The Co-Ops Study assessed the effect of reading practice, including batch size, on reader diagnostic performance in mammography within the NHSBSP and demonstrated increased specificity with increased batch size up to 40 mammograms per batch with the trend continuing in longer batches [30]. The current study, whilst it showed a trend for increasing specificity with batch size up to 50 FAST MRI scans per batch and decreasing sensitivity with increasing batch size, also demonstrated that concordance with the true outcome (as a measure of overall accuracy) tended to worsen when more than 50 scans were read within one batch. This accords with results from a study of 2,937,312 mammogram reads that demonstrated both small increases in specificity and small decreases in sensitivity for mammograms read at later positions within a batch. The authors of the study suggested that optimal batch-size for reading mammograms could be 60–70 reads per batch [31].

One possible explanation for the optimal batch size for FAST MRI (50 scans per batch) being smaller than that suggested for mammograms could be the difference in complexity between reading FAST MRI scans and mammograms. Reading FAST MRI scans in the current study could more quickly cause fatigue for readers than reading mammograms because FAST MRI reading format requires more images to be reviewed per scan than for a mammogram. However, the reading format of digital breast tomosynthesis (DBT)(2D plus stack of reconstructed slabs) has a similar complexity to that of FAST MRI (MIP plus stack of slices) and although we could find no study that reported the effect of reading batch size on the diagnostic accuracy of DBT, evidence of increasing reader fatigue during the process of reading a batch of 40 DBT scans has been reported [32].

### Literature comparison—reading times

The reading times achieved by readers in this study (56 and 78 s for Groups 1&2) were longer than times reported for NHSBSP mammogram readers to interpret mammograms (35 and 76 s [33, 34]) and about half that reported for NHSBSP mammogram readers to interpret DBT (2.81 min) [32]. However, evidence is emerging that various AI strategies may reduce reading times for DBT without affecting accuracy [35, 36]. In the future similar approaches may prove valuable for FAST MRI.

### Limitations of the current study

Readers who had previously attended FAST MRI interpretation-training had interpreted the same test set of 125 FAST MRI scans during the previous study. However, since they had not previously seen the ground truth (true outcome) of the scans in the test set at any time, and there was an average time interval of 24 months (range 17–30 months) between reading the test set in the two studies, it is unlikely that their diagnostic performance was affected by this.

The test set was read outside normal clinical practice and therefore reader performance is likely to have been subject to a laboratory effect [37].

Readers were free to self-select batch length when reading the test set assessment task. Therefore, our conclusions on optimal batch size could potentially have been confounded through self-selection bias. However, similar results were seen with the subset of readers who completed all 125 scans of the test set in a single batch (7 from Group 1 and 8 from Group 2) (Additional file 3), suggesting the effect of self-selection bias, although unquantifiable, is likely to be small.

### Implications of the research

The results of the current study demonstrate that the inclusion of immediate feedback for each scan during test set interpretation in FAST MRI reader training optimised specificity and overall diagnostic accuracy whilst maintaining high levels of sensitivity, which would be suitable for a screened population with low pre-test probability.

## Conclusions

Future trials of FAST MRI will benefit from standardising the training, assessment, and credentialing of FAST MRI readers. The diagnostic accuracy achieved at single read by NHSBSP mammogram readers in this study suggests that two-day standardised FAST MRI remote e-learning, that includes formative assessment using an enriched dataset, could form the basis for FAST MRI interpretation-training for mammogram readers who wish to participate as readers in future FAST MRI trials and clinical practice, screening populations with low pre-test probability. The credentialling of readers could be accomplished using BI-RADS benchmarks of performance [28] as cut offs for sensitivity and specificity achieved by readers in the assessment task.

### Supplementary Information

Additional file 1: The FAST MRI interpretation-training programme delivered as e-learning in the current study was adapted from a previously developed, standardised, in-person interpretation-training programme described in a previous publication. Details of the training programme have been reproduced here in line with the copyright policy of the journal in which they were previously published. (DOCX 14 KB)

Additional file 2: The specification of the FAST MRI protocol used in the current study and the composition of the assessment test-set used in the current study have been previously published. They are reproduced here in line with the copyright policy of the journal in which they were previously published. (DOCX 661 KB)

Additional file 3: Comparison graphic, included to inform discussion of our conclusions on optimal batch size The relationship of accuracy with batch size of the subset of readers who completed reading the assessment test set in a single batch of 125 FAST MRI scans is presented (for comparison with Figure 5 - the equivalent graphic for all readers). The information is presented as a graphic entitled: Changes in concordance with the true outcome (accuracy) by scan position within a batch, and by reader group*, showing only the readers that completed reading the assessment test set of 125 FAST MRI scans in a single batch (multi-level generalised mixed model using restricted cubic splines with 4 knots fitted to the rank order of FAST MRI scans read per batch). (PDF 105 KB)

## Data Availability

The dataset generated and analysed during the current study is not yet publicly available because it is currently being developed into a publicly shareable format. Instead, it is available from the corresponding author on reasonable request.

## Abbreviations

ACR: American College of Radiology
ANOVA: Analysis of variance
AUC: Area under a curve
BCSC: Breast Cancer Screening Consortium
BSIS: Breast Screening Information Service
BI-RADS: American College of Radiology’s Breast Imaging Reporting and Data System
CI: Confidence interval
DCIS: Ductal carcinoma in-situ
DOR: Diagnostic Odds Ratio
FAST MRI: First post contrast subtracted images (abbreviated breast MRI)
fpMRI: Full protocol breast Magnetic Resonance Imaging
IRAS: Integrated Research Application System for applications to the Health Research Authority and the Research Ethics Committee
ISRCTN: International Standard Randomised Controlled Trial Number (however, over the years the scope of the registry has widened beyond randomised controlled trials to include any study designed to assess the efficacy of health interventions in a human population.)
MIP: Maximum intensity projection image
MRI: Magnetic Resonance Imaging
NHSBSP: National Health Service (United Kingdom) Breast Screening Programme
PERFORMS: Personal Performance in Mammographic Screening
REC: Research Ethics Committee
ROI: Region of interest
UK: United Kingdom of Great Britain and Northern Ireland
USA: United States of America
