# Supplementary material for: Optimising the diagnostic accuracy of First post-contrAst SubtracTed breast MRI (FAST MRI) through interpretation-training: a multicentre e-learning study, mapping the learning curve of NHS Breast Screening Programme (NHSBSP) mammogram readers using an enriched dataset
Source: Breast Cancer Res. 2024 May 28;26:85. doi: 10.1186/s13058-024-01846-1 (PMC11134713; doi:10.1186/s13058-024-01846-1)
Supplement: Supplementary file 1 — Additional file 1: The FAST MRI interpretation-training programme delivered as e-learning in the current study was adapted from a previously developed, standardised, in-person interpretation-training programme described in a previous publication. Details of the training programme have been reproduced here in line with the copyright policy of the journal in which they were previously published. (DOCX 14 KB) [file 13058_2024_1846_MOESM1_ESM.docx]

## Additional file 1: Standardised FAST MRI interpretation-training programme

## The following text has been reproduced from “Jones LI, Marshall A, Elangovan P, Geach R, McKeown-Keegan S, Vinnicombe S, et al. Evaluating the effectiveness of abbreviated breast MRI (abMRI) interpretation training for mammogram readers: a multi-centre study assessing diagnostic performance, using an enriched dataset. Breast Cancer Research [Internet]. 2022 Dec 1;24(1).” (11). The following reproduction of text is in line with BMC's journals policy for open access articles which are made available under the Creative Commons Attribution (CC-BY) license (<https://www.biomedcentral.com/about/policies/reprints-and-permissions> (last accessed 13/10/2023)) © The Author(s) 2022. Open Access This article is licensed under a Creative Commons Attribution 4.0 International License:

Small group presentations on aspects of abMRI interpretation alternated with guided hands-on workstation sessions to enable learners to practice image manipulation and abMRI interpretation on the training set of 29 abMRI scans. The presentations included multiple additional illustrative examples of abMRI images depicting specific learning points. These examples were taken from MRI scans not included in either the training or test sets.

Throughout the training, mammogram readers’ prior knowledge was utilised and activated by repeated reference to similarities and differences between the two breast imaging modalities (abMRI and mammogram) and the varied appearances of cancer, and of other common breast pathologies, as displayed by each modality [Reference: Spencer J. Learning and teaching in the clinical environment How doctors teach experiential learning. Br Med J. 2003;326:591–4. https://doi. org/10.1136/bmj.326.7389.591.].

The training set was presented in batches, in the same order as in the previously reported one-to-one structured training package, as guided hands-on workstation practice during which readers could discover the ground truth at the touch of a button, giving instant feedback to aid their learning (formative assessment).
