# Supplementary material for: Optimising the diagnostic accuracy of First post-contrAst SubtracTed breast MRI (FAST MRI) through interpretation-training: a multicentre e-learning study, mapping the learning curve of NHS Breast Screening Programme (NHSBSP) mammogram readers using an enriched dataset
Source: Breast Cancer Res. 2024 May 28;26:85. doi: 10.1186/s13058-024-01846-1 (PMC11134713; doi:10.1186/s13058-024-01846-1)

## Additional file 2: Specification of the FAST MRI protocol and composition of the assessment test-set

###

### FAST MRI protocol

The following text has been reproduced from: ﻿Jones LI, Geach R, Harding SA, Foy C, Taylor V, Marshall A, et al. “Can mammogram readers swiftly and effectively learn to interpret first post-contrast acquisition subtracted (FAST) MRI, a type of abbreviated breast MRI?: a single centre data-interpretation study”. **Br J Radiol 2019**; 92: 20190663 (13). The British Institute of Radiology (BIR) holds the copyright for the original article. The following reproduction of the text is in line with BIR policy: <https://www.birpublications.org/page/permissions#:~:text=the%20original%20source.-,Are%20you%20the%20author%20of%20the%20original%20article%3F,free%20of%20charge%20providing%20they%20cite%20the%20original%20source%20article.,-BJR%7Ccase%20reports> (last accessed 13/10/2023):

Breast MRI protocol

All MRI examinations in the dataset were originally acquired on either a Philips (Amsterdam, Netherlands) Ingenia 1.5T or a Philips Ingenia 3T scanner. The breast coils used were dStream Breast seven-channel coils. The paramagnetic contrast agent used was gadobutrol 1.0 mmol ml−1 and the dose administered was 0.1 ml gadobutrol per kg body weight. The dynamic sequence used (from which the dataset’s FAST MRI images were obtained through post-processing) was dyn_eTHRIVE (Axial 3D T1 fast field echo (FFE), TR/TE 5.1/2.8 with 10 degree flip angle and SPAIR Power two fat suppression). Post-contrast scan commenced contemporaneous with the commencement of contrast injection (average duration 1.08 minutes)). Since the images used in the current study were originally acquired in 2015 and then later reprocessed and anonymised for the study, the acquisition protocol conformed to our own centre’s standard. This differed from Kuhl’s description of FAST MRI (11) as follows:

1. The breasts were not compressed during MR acquisition.
2. (ii) The T1 images of the dynamic study that were used to form the subtracted images were fat-suppressed (dyn_eTHRIVE).

The MRI scans performed for a screening indication were performed during day 6–16 of the woman’s menstrual cycle, but those performed post cancer diagnosis were performed promptly without reference to the woman’s menstrual cycle.

The MRI studies were copied, anonymised and allocated study identifiers chronologically for the date they were acquired and, as a consequence, normal and abnormal scans were presented to the readers in an unpredictable order. They were then reduced to comprise simply those MR sequences that would have been obtained if they had originally been acquired as a FAST MRI, displayed as an axial maximum intensity projection image (MIP), and also as a stack of axial slices (slice stack) of the first post-contrast-subtracted images from the dynamic series of the breast MRI examination. This process was performed by two of the research team who were not subsequently part of either of the two reading groups. These subtracted images alone comprised the FAST MRI scans interpreted by the readers.

### Composition of the assessment test set of FAST MRI scans used in the study


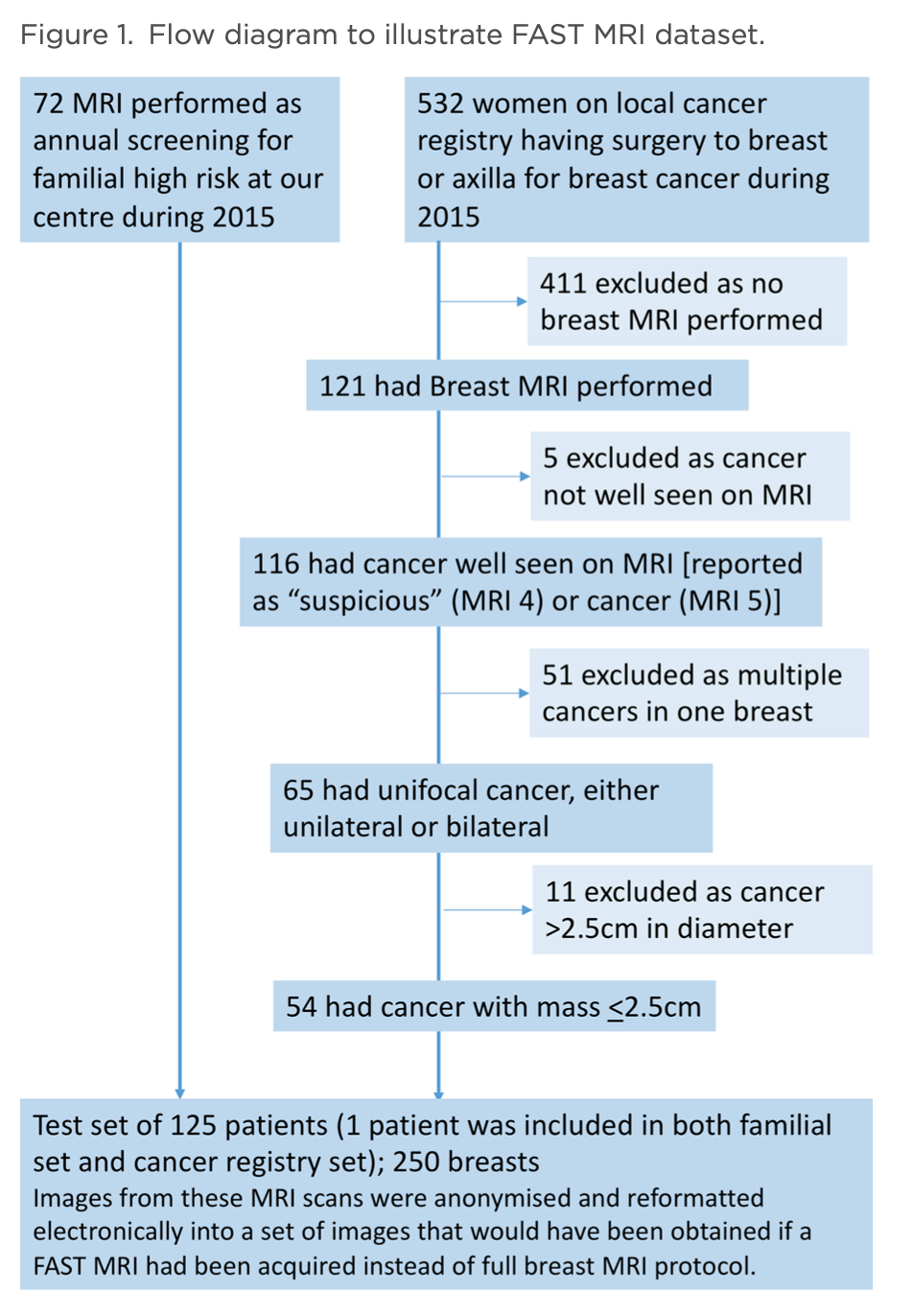
The following figure and table have been reproduced from: ﻿Jones LI, Geach R, Harding SA, Foy C, Taylor V, Marshall A, et al. “Can mammogram readers swiftly and effectively learn to interpret first post-contrast acquisition subtracted (FAST) MRI, a type of abbreviated breast MRI?: a single centre data-interpretation study”. **Br J Radiol 2019**; 92: 20190663 (13). The British Institute of Radiology (BIR) holds the copyright for the original article. The following reproduction of the text is in line with BIR policy: <https://www.birpublications.org/page/permissions#:~:text=the%20original%20source.-,Are%20you%20the%20author%20of%20the%20original%20article%3F,free%20of%20charge%20providing%20they%20cite%20the%20original%20source%20article.,-BJR%7Ccase%20reports> (last accessed 13/10/2023):

##
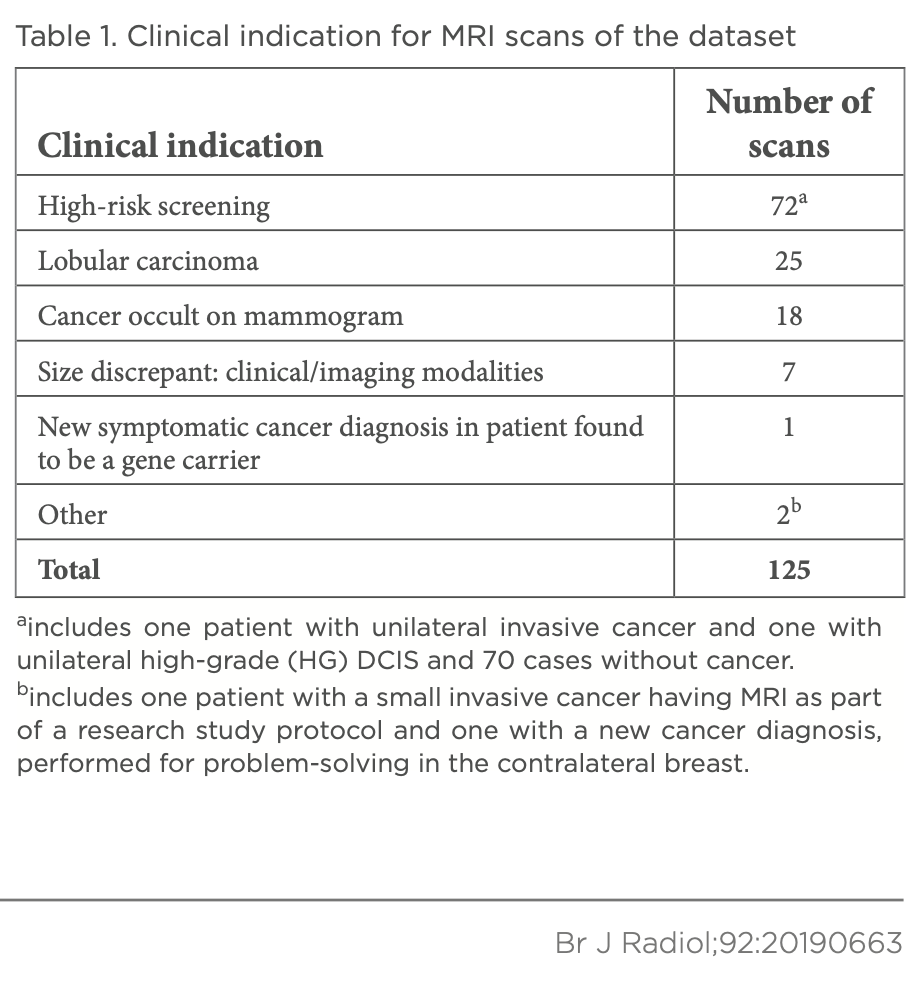

Supplement: Supplementary file 2 — Additional file 2: The specification of the FAST MRI protocol used in the current study and the composition of the assessment test-set used in the current study have been previously published. They are reproduced here in line with the copyright policy of the journal in which they were previously published. (DOCX 661 KB) [file 13058_2024_1846_MOESM2_ESM.docx]
