# Supplementary material for: Optimising the diagnostic accuracy of First post-contrAst SubtracTed breast MRI (FAST MRI) through interpretation-training: a multicentre e-learning study, mapping the learning curve of NHS Breast Screening Programme (NHSBSP) mammogram readers using an enriched dataset
Source: Breast Cancer Res. 2024 May 28;26:85. doi: 10.1186/s13058-024-01846-1 (PMC11134713; doi:10.1186/s13058-024-01846-1)
Supplement: Supplementary file 3 — Additional file 3: Comparison graphic, included to inform discussion of our conclusions on optimal batch size The relationship of accuracy with batch size of the subset of readers who completed reading the assessment test set in a single batch of 125 FAST MRI scans is presented (for comparison with Figure 5 - the equivalent graphic for all readers). The information is presented as a graphic entitled: Changes in concordance with the true outcome (accuracy) by scan position within a batch, and by reader group*, showing only the readers that completed reading the assessment test set of 125 FAST MRI scans in a single batch (multi-level generalised mixed model using restricted cubic splines with 4 knots fitted to the rank order of FAST MRI scans read per batch). (PDF 105 KB) [file 13058_2024_1846_MOESM3_ESM.pdf]

Additional file 3: Comparison graphic, included to inform discussion of our conclusions on optimal batch size

Changes in concordance with the true outcome (accuracy) by scan position within a batch, and by reader group*, showing only the readers that completed reading the assessment test set of 125 FAST MRI scans in a single batch (multi-level generalised mixed model using restricted cubic splines with 4 knots fitted to the rank order of FAST MRI scans read per batch)

There were 15 readers that did this (7 from group 1 and 8 from group 2).

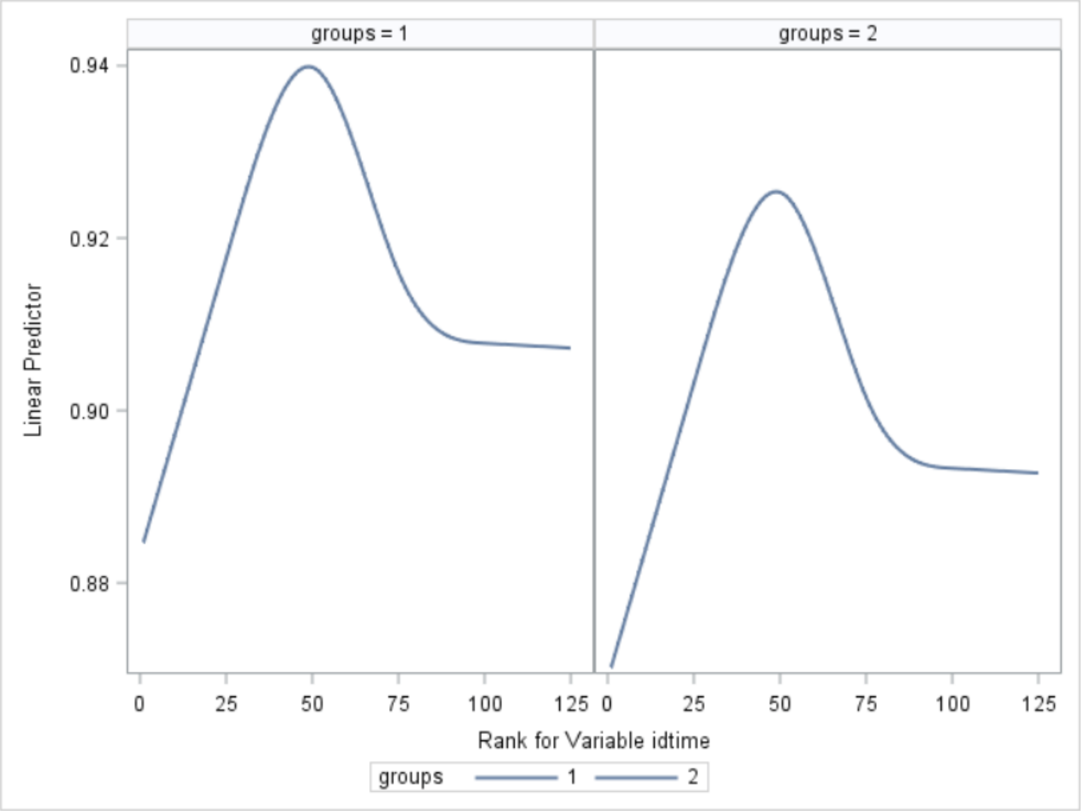

* Reader group: group 1 = experience of fpMRI interpretation in their usual clinical practice, group 2 = no previous experience of breast MRI interpretation in their clinical practice
